# Comparing visual outcomes of nAMD treatment during and after the COVID-19 restrictions period

**DOI:** 10.1371/journal.pone.0323253

**Published:** 2025-05-07

**Authors:** Mike Y. Chen, Jeanette Du, Brian K. Do, Mohsin H. Ali

**Affiliations:** 1 Georgetown University School of Medicine, Washington, DC, United States of America; 2 Retina Group of Washington, Chevy Chase, Maryland, United States of America; Sanmenxia Central Hospital, Henan University of Science and Technilogy, CHINA

## Abstract

**Main objective:**

Compare treatment outcomes of newly diagnosed neovascular age-related macular degeneration (nAMD) during and after the COVID-19 restrictions.

**Methods:**

This retrospective study at the Retina Group of Washington analyzed nAMD patients treated with anti-VEGF therapy with ≥ 12 months of follow-up. Two groups were identified: 258 subjects diagnosed between March 2020-March 2022 (Group 1) and 376 subjects diagnosed after (Group 2). Primary outcomes were 12-month and final best-corrected visual acuity (BCVA), and number of injections in the first 12 months.

**Results:**

Initial mean BCVA was 20/71 and 20/68 in Group 1 and Group 2, with median BCVA of 20/60 and 20/50, respectively. At 12 months, mean BCVA improved to 20/65 and 20/54 in Group 1 and Group 2, respectively (p = 0.086). Final mean BCVA was 20/76 for Group 1 and 20/58 for Group 2 (p = 0.010). The mean change in LogMAR BCVA from the time of conversion to last follow-up was + 0.03 for Group 1 and -0.08 for Group 2 (p = 0.007). Group 1 had fewer injections in the first year of therapy (8.67 vs. 9.21, p = 0.004). 38.8% of Group 1 reached BCVA ≥20/40 at 12 months, versus 48.9% for Group 2 (p = 0.011).

**Conclusion:**

Patients diagnosed during the COVID-19 restrictions period had worse visual outcomes than those diagnosed thereafter. Multiple factors, including, but not limited to reduced treatment frequency, likely contributed to worse visual outcomes.

## Introduction

Neovascular age-related macular degeneration (nAMD) remains a leading cause of visual impairment among the elderly population, affecting over 200 million people worldwide [1,2]. The introduction of anti-vascular endothelial growth factor (anti-VEGF) therapies has significantly transformed the treatment landscape, markedly improving both prognosis and quality of life for patients with nAMD [3]. Despite these advances, the efficacy of treatment regimens is highly dependent on adherence to frequent and long-term treatment schedules [4]. The necessity for consistent management can pose substantial challenges in the face of global health crises like the COVID-19 pandemic, which widely disrupted outpatient care delivery [5].

The advent of the COVID-19 pandemic drastically altered healthcare delivery around the world. Preventive measures and reallocation of resources led to delayed care and reduced access to treatment for many non-communicable diseases, including nAMD [6]. Such disruptions pose significant risks to the visual outcomes in nAMD patients, as timely and regular treatments are critical to prevent vision loss. Indeed, research during the pandemic period indicated a notable decline in the number of ophthalmology visits [7]. Other studies have also documented worsened visual outcomes, decreased intravitreal anti-VEGF injections, and increased number of submacular hemorrhages among patients with nAMD during this period [6,8–13].

Our study aims to fill a gap in the current literature concerning the impact of the pandemic on the management and visual outcomes of new nAMD cases. This study investigates how treatment frequency and visual outcomes of patients at a large retina practice may have been affected by the COVID-19 pandemic by comparing those diagnosed during the restricted COVID period to those diagnosed after this period. Through this comparative analysis, we seek to understand the extent of pandemic-related disruptions in nAMD treatment and their implication for patient care, with the ultimate goal of enhancing treatment strategies during ongoing and future health crises.

## Materials and methods

### Study design

This is a retrospective cohort study conducted at the Retina Group of Washington (RGW). Institutional Review Board (IRB) approval was granted to conduct the study. Authors MYC and JD had access to information that could identify individual participants during and after data collection. Electronic health records (EHR) were reviewed on May 23, 2024 to identify two groups. Adult subjects with nAMD treated with intravitreal anti-VEGF therapy and at least 12 months of follow-up were included. Medical records that carried an ICD10 diagnosis code for bilateral dry AMD (H35.3131-3) and subsequent records with an ICD10 code for nAMD (H35.32xx) concurrent with the procedure code for intravitreal injection (67028) were used to identify subjects that were established with RGW and followed for dry AMD. Medical records that carried an ICD10 diagnosis code for nAMD with concurrent new patient office visit CPT codes (92004, 99204) and procedure code for intravitreal injection (67028) were also used to identify new patients who were referred to RGW by optometrists or general ophthalmologists for nAMD. Group 1 subjects were patients with newly diagnosed nAMD during the COVID-19 restrictions period, defined as between March 15, 2020, and March 31, 2022. These dates were selected based on key pandemic milestones: March 15, 2020 marks the onset of widespread state-implemented shutdowns, and March 2022 corresponds to the lifting of the last statewide indoor mask mandate [14]. Group 2 subjects were patients with newly diagnosed nAMD after March 31, 2020. Patient and public involvement was not appropriate for this study, which analyzed pre-existing clinical data without additional direct patient interaction or data collection. All subjects were cases of new neovascular disease of the first eye.

### Exclusion criteria

Subjects were excluded if they had a history of intravitreal anti-VEGF therapy in either eye prior to the diagnosis of nAMD, and non-AMD related causes of severe vision loss in the study eye, such as optic neuropathy, posterior uveitis, endophthalmitis, retinal detachment, open globe injury, amblyopia, macular hole, retinal vein occlusion, and retinal artery occlusion. Patients treated for AMD or a comorbid diagnosis with other classes of intravitreal medication, such as complement inhibitors or steroids, during the follow-up period were also excluded.

### Data collection

Data on patient demographics, comorbidities, clinical exam findings, and intravitreal injections were collected. The primary outcome measures were BCVA at 12 months and at the last follow-up following the diagnosis of nAMD, and the number of injections given in the first 12 months of treatment. The type of anti-VEGF injection was also recorded. The presence of submacular hemorrhage on clinical exam upon diagnosis of neovascular conversion was noted.

### Statistical analysis

Clinical data were compared using the Student’s t-test for numerical variables and the χ2-test with odds ratio (OR) and 95% confidence interval (CI) for categorical variables. For outcome measures, BCVA was converted to the logarithm of the minimal angle of resolution (LogMAR) scale for statistical analysis. Multivariate linear regression and multivariate logistic regression analyses were employed to assess the influence of various predictors on BCVA at various time points. At the time of neovascular conversion, predictors included group designation, birth sex, laterality, phakic status, submacular hemorrhage, and age. For BCVA assessments at 12 months and at the last follow-up, the models also included the average follow-up time, BCVA at the time of neovascular conversion, and number of injections given in the first 12 months of therapy in addition to the initial predictors. For multivariate linear regression models, LogMAR BCVA was converted to its decimal equivalent (equation: 10^(-LogMAR BCVA)) to help normalize the distribution of BCVA values and stabilize variance across the range of predictions, enhancing the model fit. A p-value ≤0.05 was established *a priori* as the rejection criterion for all analyses.

## Results

### Baseline patient and eye characteristics

Group 1 was composed of 258 eyes that converted to nAMD during the COVID-19 restrictions period. Group 2 was composed of 376 eyes that converted to nAMD after the restrictions period. All subjects were diagnosed with new nAMD between March 2020 and March 2023. Average follow-up time after starting anti-VEGF injections was 28.1 months for Group 1 and 17.3 months for Group 2 (p < 0.001). Mean age, proportion of patients presenting with submacular hemorrhage, and average LogMAR BCVA at the time of neovascular conversion were similar for both groups (p = 0.417; p = 0.691; p = 0.634, respectively). Baseline eye characteristics are detailed in Table 1.

**Table 1:** Baseline demographics and clinical characters of patients from restricted (Group 1) and non-restricted (Group 2) COVID-19 period with newly diagnosed nAMD of the first eye.

Characteristics	Group 1 N = 258	Group 2 N = 376	Odds ratio (95% CI)	P value
Mean years of age at presentation, No. (SD)	79.97 (8.9)	79.39 (8.7)	n/a	0.417^a^
Birth Sex, No. of Female (%)	164 (63.6)	246 (65.4)	0.92 (0.66-1.28)	0.630^b^
Laterality, No. of OD (%)	138 (53.5)	196 (52.7)	1.05 (0.77-1.45)	0.736^b^
Phakic status, No. (%)	100 (38.9)	143 (38.0)	1.03 (0.74-1.43)	0.853^b^
Submacular hemorrhage at time of conversion, No. (%)	40 (15.5)	54 (14.4)	0.91 (0.59-1.42)	0.691^b^
Mean BCVA in LogMAR at time of conversion (SD)	0.55 (0.5)	0.53 (0.5)	n/a	0.634^a^
BCVA ≥20/40 at time of conversion, No. (%)	70 (27.1)	114 (30.3)	1.17 (0.82-1.66)	0.385^b^
BCVA ≤20/70 at time of conversion, No. (%)	104 (40.3)	127 (33.8)	0.76 (0.54-1.05)	0.093^b^
BCVA ≤20/200 at time of conversion, No. (%)	41 (15.9)	59 (15.7)	0.99 (0.64-1.52)	0.946^b^
No. of anti-VEGF injections in 12 months, mean (SD)	8.67 (2.3)	9.21 (2.2)	n/a	0.004^a^
No. of anti-VEGF injections in total, mean (SD)	16.6 (6.5)	11.9 (3.9)	n/a	<0.001^a^
Mean total follow-up in months, No. (SD)	28.43 (6.4)	17.35 (3.2)	n/a	<0.001^a^

^a^Student’s T test;

^b^χ^2^-test; BCVA = best-corrected visual acuity; CI = confidence interval; nAMD = neovascular age-related macular degeneration; SD = standard deviation.

### Outcomes

Average BCVA at various time points over the follow-up period are detailed in Table 2. At 12 months, mean BCVA improved to 0.51 (20/65) for Group 1 and 0.43 (20/54) for Group 2 (p = 0.086). At the last follow-up, BCVA was 0.58 (20/76) for Group 1 and 0.46 (20/58) for Group 2 (p = 0.010). The proportion of patients with BCVA ≥ 20/40 at 12 months after initiation of therapy was 38.8% in Group 1 compared to 48.9% in Group 2 (p = 0.011). At last follow-up, the proportion of patients with BCVA ≥ 20/40 was 35.7% in Group 1 compared to 46.3% for Group 2 (p = 0.008). The proportion of patients with BCVA ≤ 20/70 and ≤ 20/200 at the last follow-up was 35.3% and 20.5% in Group 1 compared to 25.0% and 13.8% in Group 2, respectively (p = 0.005; p = 0.026). Group 1 had, on average, a worse mean BCVA at the last follow-up compared to the time of conversion, whereas Group 2 had a better mean BCVA.

**Table 2:** Outcomes of patients from restricted (Group 1) and non-restricted (Group 2) COVID-19 period with newly diagnosed nAMD of the first eye.

	Group 1, N = 258	Group 2, N = 376	Difference [95% CI]	P value	Odds ratio (95% CI)
Mean BCVA at time of conversion to nAMD (SD)	0.55 (0.5)	0.53 (0.5)	-0.02 [-0.10, 0.06]	0.634^a^	n/a
Mean BCVA at 6 months (SD)	0.49 (0.5)	0.43 (0.5)	-0.06 [-0.14, 0.02]	0.165^a^	n/a
Mean BCVA at 12 months (SD)	0.51 (0.5)	0.43 (0.5)	-0.08 [-0.16, 0.01]	0.086^a^	n/a
Mean BCVA at last follow up (SD)	0.58 (0.6)	0.45 (0.6)	-0.12 [-0.22, -0.03]	0.010^a^	n/a
BCVA ≥20/40 at 12 months, No. (%)	100 (38.8)	184 (48.9)	n/a	0.011^b^	1.51 (1.10-2.09)
BCVA ≥20/40 at last follow up, No. (%)	92 (35.7)	174 (46.3)	n/a	0.008^b^	1.55 (1.12-2.15)
BCVA ≤20/70 at 12 months, No. (%)	76 (29.5)	88 (23.4)	n/a	0.089^b^	0.73 (0.51-1.05)
BCVA ≤20/70 at last follow up, No. (%)	91 (35.3)	94 (25.0)	n/a	0.005^b^	0.61 (0.43-0.86)
BCVA ≤20/200 at 12 months, No. (%)	44 (17.1)	49 (13.0)	n/a	0.162^b^	0.73 (0.47-1.13)
BCVA ≤20/200 at last follow up, No. (%)	53 (20.5)	52 (13.8)	n/a	0.026^b^	0.62 (0.41-0.95)
Mean changes in LogMAR BCVA at 12 months to time of conversion (SD)	-0.04 (0.4)	-0.01 (0.4)	n/a	0.092^a^	n/a
Mean changes in LogMAR BCVA at last follow-up to time of conversion (SD)	0.03 (0.5)	-0.08 (0.4)	n/a	0.007^a^	n/a

^a^Student’s T test;

^b^χ^2^-test; BCVA = best-corrected visual acuity; CI = confidence interval; nAMD = neovascular age-related macular degeneration; SD = standard deviation.

The total number of anti-VEGF injections given in the first 12 months of therapy was 8.7 ± 2.3 for Group 1 and 9.2 ± 2.2 for Group 2, which was significantly fewer for Group 1 (p = 0.004). In contrast, the total number of anti-VEGF injections given from initiation to the last follow-up was 16.6 ± 6.5 for Group 1 and 11.9 ± 3.9 for Group 2 (p < 0.001). Types of injections given over this period included bevacizumab (12.6% for Group 1 vs. 14.6% for Group 2, p = 0.369), aflibercept 2mg (52.2% vs. 58.6%, p = 0.045), aflibercept 8mg (1.9% vs. 4.9%, p < 0.001), ranibizumab (28.4% vs. 13.4%, p < 0.001), and faricimab (4.8% vs. 8.6%, p = 0.002). The most frequent type of injection given during the first 12 months of therapy and through the last follow-up was aflibercept 2 mg, which accounted for 50.2% and 50.9% of injections given for Group 1 and 63.5% and 60.2% of injections given for Group 2 patients, respectively.

### Multivariate regression analyses

In a multivariate linear regression analysis of all patients, higher baseline BCVA and younger mean age at the time of neovascular conversion were significant positive predictors of the transformed BCVA at 12 months, holding constant all other variables in the model including birth sex, laterality, phakic status, total number of injections within the first 12 months of therapy, total follow-up time in months, group designation, transformed baseline BCVA, mean age, and submacular hemorrhage at the time of conversion (p < 0.001, Table 3). Higher baseline BCVA and younger mean age at the time of neovascular conversion remained as significant positive predictors of the transformed BCVA at the last follow-up, holding constant all other variables (p < 0.001 and p = 0.001, respectively, Table 3). Group 1 was a significant negative predictor of the transformed BCVA at the last follow-up, controlling for other variables including birth sex, laterality, phakic status, total number of injections within the first 12 months of therapy, total follow-up time in months, transformed baseline BCVA, mean age, and submacular hemorrhage at the time of conversion (p = 0.027, Table 3). Group designation was not a significant predictor of transformed BCVA at time of neovascular conversion controlling for other variables including birth sex, age, laterality, phakic status, submacular hemorrhage, and group designation (Table 3).

**Table 3:** Standard Least Square Model of BCVA at different time points.

Transformed BCVA at time of neovascular conversion						
Term	Estimate	Std Error	t Ratio	Prob>|t|	Lower 95%	Upper 95%
Intercept	1.003	0.109	9.24	<0.001	0.790	1.217
Sex [F]	0.004	0.011	0.34	0.730	-0.018	0.025
Laterality [OD]	0.024	0.010	2.33	**0.020**	0.004	0.044
Lens status [Phakic]	-0.025	0.012	-2.00	**0.046**	-0.050	0.000
Submacular hemorrhage [No]	0.066	0.015	4.55	**<0.001**	0.038	0.095
Age at time of conversion	-0.008	0.001	-5.83	**<0.001**	-0.011	-0.005
Groups [Group 1]	-0.014	0.010	-1.3	0.193	-0.034	0.007
**Transformed BCVA at 12 months**						
**Term**	**Estimate**	**Std Error**	**t Ratio**	**Prob>|t|**	**Lower 95%**	**Upper 95%**
Intercept	0.590	0.129	4.57	<0.001	0.336	0.843
Sex [F]	-0.004	0.010	-0.38	0.704	-0.023	0.016
Laterality [OD]	0.014	0.009	1.53	0.128	-0.004	0.033
Lens status [Phakic]	-0.005	0.011	-0.43	0.671	-0.027	0.017
Submacular hemorrhage [No]	0.019	0.013	1.43	0.154	-0.007	0.046
Transformed BCVA at time of neovascular conversion	0.652	0.036	17.90	**<0.001**	0.580	0.723
Total follow up in months	0.001	0.002	0.45	0.653	-0.003	0.005
Total number of injections in the first 12 months	0.005	0.004	1.13	0.260	-0.004	0.013
Age at time of conversion	-0.005	0.001	-4.19	**<0.001**	-0.008	-0.003
Groups [Group 1]	-0.024	0.015	-1.63	0.103	-0.053	0.005
**Transformed BCVA at last follow-up**						
**Term**	**Estimate**	**Std Error**	**t Ratio**	**Prob>|t|**	**Lower 95%**	**Upper 95%**
Intercept	0.469	0.133	3.52	<0.001	0.207	0.731
Sex [F]	0.002	0.010	0.22	0.823	-0.018	0.022
Laterality [OD]	0.014	0.010	1.48	0.140	-0.005	0.033
Lens status [Phakic]	0.005	0.012	0.45	0.650	-0.018	0.028
Submacular hemorrhage [No]	0.014	0.014	0.97	0.331	-0.014	0.041
Transformed BCVA at time of neovascular conversion	0.652	0.038	17.32	**<0.001**	0.578	0.726
Total follow up in months	0.001	0.002	0.57	0.568	-0.003	0.005
Total number of injections in the first 12 months	0.007	0.004	1.55	0.123	-0.002	0.015
Age at time of conversion	-0.004	0.001	-3.30	**0.001**	-0.007	-0.002
Groups [Group 1]	-0.034	0.015	-2.22	**0.027**	-0.063	-0.004

LogMAR BCVA was converted to its decimal equivalent to help normalize the distribution of BCVA values and stabilize variances. Model Fit for BCVA at time of neovascular conversion: R-square = 0.092, adjusted R-square = 0.83, F-statistic = 10.6 (6, 627), N = 634. Model Fit for BCVA at 12: R-square = 0.419, adjusted R-square = 0.411, F-statistic = 50.0 (9, 624), N = 634. Model Fit for BCVA at last follow-up: R-square = 0.400, adjusted R-square = 0.391, F-statistic = 46.2 (9, 624), N = 634. F = Female; OD = oculus dexter (right eye); Std Error = standard error; Lower 95% and Upper 95% = confidence interval.

In multivariate nominal logistic analyses, Group 1 remains as a negative significant predictor of the proportion of patients with BCVA ≥ 20/40 at 12 months and at last follow-up, controlling for other variables including birth sex, laterality, phakic status, total number of injections within the first 12 months of therapy, total follow-up time in months, mean age, submacular hemorrhage, and the proportion of patients with BCVA ≥ 20/40 at the time of conversion (p = 0.005 and 0.002, respectively).

### Subgroup analysis

In a subgroup analysis of patients who received aflibercept 2mg only, BCVA at last follow-up, proportion of patients with BCVA ≥20/40 at 12 months and at last follow-up remained significantly different between the groups (Table 4). In multivariate linear regression analysis, Group 1 remained as a negative significant predictor of transformed BCVA at the last follow-up, controlling for other variables including birth sex, laterality, phakic status, total number of injections within the first 12 months of therapy, total follow-up time in months, transformed baseline BCVA, mean age, and submacular hemorrhage at the time of conversion (p = 0.012).

**Table 4:** Outcomes of patients from restricted (Group 1) and non-restricted (Group 2) COVID-19 period with newly diagnosed nAMD of the first eye who have received aflibercept 2mg injections only.

	Group 1, N = 67	Group 2, N = 97	Difference [95% CI]	P value	Odds ratio (95% CI)
Mean BCVA at time of conversion (SD)	0.61 (0.5)	0.59 (0.6)	-0.02 [-0.20, 0.15]	0.795^a^	n/a
Mean BCVA at 6 months (SD)	0.52 (0.6)	0.45 (0.6)	-0.08 [-0.26, 0.10]	0.410^a^	n/a
Mean BCVA at 12 months (SD)	0.57 (0.6)	0.42 (0.6)	-0.15 [-0.33, 0.04]	0.125^a^	n/a
Mean BCVA at last follow up (SD)	0.67 (0.7)	0.42 (0.5)	-0.24 [-0.45, -0.04]	**0.020** ^a^	n/a
BCVA ≥20/40 at time of conversion, No (%)	12 (17.9)	28 (28.9)	n/a	0.104	1.86 (0.87-3.99)
BCVA ≥20/40 at 12 months, No. (%)	24 (35.8)	53 (54.6)	n/a	**0.017** ^b^	2.16 (1.14-4.09)
BCVA ≥20/40 at last follow up, No. (%)	22 (33.8)	50 (51.6)	n/a	**0.017** ^b^	2.18 (1.14-4.16)
BCVA ≤20/70 at time of conversion, No (%)	27 (40.3)	37 (38.1)	n/a	0.781^b^	0.91 (0.48-1.73)
BCVA ≤20/70 at 12 months, No. (%)	23 (34.3)	20 (20.6)	n/a	**0.050** ^b^	0.50 (0.25-1.00)
BCVA ≤20/70 at last follow up, No. (%)	26 (38.8)	21 (21.5)	n/a	**0.017** ^b^	0.44 (0.22-0.87)
BCVA ≤20/200 time of conversion, No. (%)	15 (22.4)	19 (19.6)	n/a	0.664^b^	0.84 (0.39-1.81)
BCVA ≤20/200 at 12 months, No. (%)	14 (20.9)	14 (14.4)	n/a	0.278^b^	0.64 (0.28-1.45)
BCVA ≤20/200 at last follow up, No. (%)	16 (23.9)	13 (13.4)	n/a	0.084^b^	0.49 (0.22-1.11)
No. of anti-VEGF injections in 12 months, mean (SD)	8.25 (2.2)	8.23 (2.2)	n/a	0.939^a^	n/a
Mean total follow-up in months, No. (SD)	27.8 (7.1)	17.4 (3.5)	n/a	**<0.001** ^a^	n/a

^a^Student’s T test;

^b^χ^2^-test; BCVA = best-corrected visual acuity; CI = confidence interval; nAMD = neovascular age-related macular degeneration; SD = standard deviation.

## Discussion

This study provides important insights into the management and outcomes of patients newly diagnosed with nAMD during the restricted period of COVID-19 pandemic. Despite comparable baseline characteristics such as initial BCVA and rate of submacular hemorrhage, the pandemic was associated with poorer visual outcomes for patients diagnosed during the restricted period (Group 1) compared to those diagnosed after this period (Group 2). The impact of the pandemic may have influenced both clinical outcomes and the frequency of treatment.

Patients in Group 1 had worse visual outcomes at 12 months and last follow-up compared to Group 2, with a lower proportion achieving a BCVA ≥ 20/40 and a higher proportion with BCVA ≤ 20/70. Group 2 had a better change in LogMAR BCVA at the last follow-up compared to the time of conversion than Group 1. Importantly, these differences emerged despite initial similarity in baseline clinical features at the time of neovascular conversion, including BCVA and the rate of submacular hemorrhage. Our study found no differences in the rate of submacular hemorrhage at the time of neovascular conversion, which contrasts with the report by Roman *et al.* that found increased incidence of submacular hemorrhage in nAMD patients during COVID-19 [13]. However, theirs was a report from Italy on the early months of the pandemic, specifically comparing lockdowns in March and April of 2020 to control months in 2019. Thus, this difference is likely due to the variation in the study period. Our findings suggest that the broader pandemic period may not have been associated with delayed diagnosis of nAMD, but could have impacted the optimal management of neovascular disease.

We observed a reduced number of intravitreal injections, with a lower average number of injections in the first 12 months in Group 1 compared to Group 2. This could reflect challenges in maintaining routine injection schedules during the pandemic. Indeed, many studies have documented the significant impact of COVID-19 on treatment schedules and visual outcomes of patients with nAMD. For instance, *Borrelli et al.* reported a decline in outpatient visits and intravitreal injections in Italy, with significantly worsened visual acuity in patients due to delayed follow-up [10]. Similar findings were observed in Turkey, France, and Switzerland, where studies focused on the initial months of the lockdown period in early 2020 and found delays in anti-VEGF treatment and associated worsened visual outcomes [6,15,16]. All of the mentioned studies included nAMD patients who had already established care and had begun anti-VEGF therapy. Our study is unique in investigating newly diagnosed nAMD patients during a prolonged period following the initial lockdown, suggesting that the impact of the pandemic on patients newly diagnosed with nAMD may continue to affect treatment frequency two years post-lockdown.

Our multivariate regression analyses showed that age was negatively correlated with final visual acuity, while higher baseline visual acuity correlated with better outcomes. These findings are consistent with known factors affecting nAMD outcomes including age and initial BCVA [17–21]. Interestingly, while newly diagnosed nAMD during the restricted COVID period was not a predictor of BCVA at 12 months, it was a predictor of the final BCVA and was significantly associated with the proportion of patients achieving BCVA ≥ 20/40 at 12 months and at last follow-up, even after adjusting for potential confounding variables including total follow-up time. This suggests that nAMD diagnosis during the restricted COVID period was associated with poorer outcomes within the first year of treatment and may affect visual outcomes longer term. This finding contrasts with some reports indicating no difference in BCVA during the COVID period. *Nassisi et al.* reported that during the initial lockdown months in Italy, about 73% of nAMD patients had delayed treatment, yet there was no significant difference in BCVA after 6 months compared to those who maintained their treatment schedules during the same period [12]. Similarly, a study from Sweden showed that the BCVA of treatment-naïve nAMD patients at 1 year follow-up was comparable between patients from the early months of the pandemic and those from pre-COVID times [22]. Furthermore, *Barequet et al.* from Israel examined nAMD patients from 2019 (pre-COVID), 2020 (COVID), and 2021 (post-COVID) and found no difference in the final BCVA across these groups [23]. The variations in findings are likely due to differences in study period, follow-up time, and geographic locations.

The main limitation of this study is its retrospective design, which inherently limits control over confounding variables. As a result, factors other than those measured could influence the timely diagnosis, treatment, and outcomes of nAMD. Additionally, although our study was conducted at a large retina practice across seventeen office locations in Virginia, Maryland, and Washington, D.C., the findings may not be entirely generalizable to other geographic locations or practice settings. The limitation is pertinent given the varied levels of pandemic restrictions in different regions which could influence patient management strategies and outcomes in different regions. Further, our analysis did not include data on potential adjustments in treatment regimens, such as switching between anti-VEGF agents in cases of treatment resistance. Our study also did not account for possible variations in drug efficacy. Some agents, such as aflibercept 2mg, were more commonly used in Group 2, which could have contributed to better visual outcomes independently of treatment frequency. However, in our subgroup analysis with patient received aflibercept 2mg only, Group 2 continued to show better visual outcomes at month 12 and at last follow-up, despite having similar BCVA at time of conversion and after controlling for total length of follow-up. Nevertheless, accounting for these treatment dynamics in future studies could provide further insights into optimizing management strategies for nAMD. Future research should also consider multi-center studies and the evaluation of alternative delivery models such as telehealth services, which have been shown to improve follow-up rates and facilitate earlier detection of nAMD, thus enhancing patient care during health crises [24]. Further investigation into telehealth’s ongoing impact on treatment frequency, follow-up rates, and visual outcomes in nAMD patients would also be valuable.

In conclusion, our study corroborates the notion that healthcare disruptions during the COVID-19 pandemic may have affected the care of patients newly diagnosed with nAMD. The restricted COVID period was associated with poorer visual outcomes in the setting of reduced frequency of anti-VEGF injections. The impact of these disruptions may affect the likelihood of achieving optimal visual outcomes within the first year of treatment. Our findings emphasize the importance of developing adaptable strategies to maintain effective patient care in the face of external challenges posed by a global pandemic.

## Supporting information

S1 File Minimum data set. (XLSX)

## Abbreviations

nAMD: neovascular age-related macular degeneration
EHR: electronic health record
VEGF: vascular endothelial growth factor
BCVA: best-correct visual acuity.
